# Addressing Global Gaps in Mammography Screening for Improved Breast Cancer Detection: A Review of the Literature

**DOI:** 10.7759/cureus.66198

**Published:** 2024-08-05

**Authors:** Shreya Naik, Albert P Varghese, Syed Asrar Ul Haq Andrabi, Suhas Tivaskar, Anurag Luharia, Gaurav V Mishra

**Affiliations:** 1 Radiology, Datta Meghe Institute of Higher Education and Research, Wardha, IND

**Keywords:** breast imaging guidelines, mammography screening, breast cancer, quality assurance, quality control

## Abstract

Breast cancer is the second most common cancer globally, with 2.3 million new cases annually, constituting 11.6% of all cancer cases. It is also the fourth leading cause of cancer deaths, claiming 670,000 lives a year. This high incidence of breast cancer morbidity worldwide has increased the urgent need for standardized and adequate screening methods, including clinical breast examination, self-breast examination, and mammography screening tests for non-symptomatic individuals. Mammography is considered the gold standard for breast cancer screening, with early randomized control trials showing significant reductions in mortality rates in women aged 50 and over (International Agency for Research on Cancer and American College of Radiology). Despite this, discrepancies in mammography practices across different healthcare settings regarding adherence to international standards raise concerns. A comprehensive review of the vast literature looking at the practices and norms of mammography screening worldwide highlighted several domains that present limitations to screening. These include epidemiological data deficits, lack of educational training offered to radiographers and varied image quality indices, exposure technique, method of breast compression, dose calculation, reference levels, screening frequency intervals, and diverse distribution of resources, particularly in developing countries. These factors shed light on the substantial discrepancies in the implementation and efficacy of screening programs, underscoring the necessity for future research endeavors to collaborate in creating coherent, standardized, evidence-based guidelines. Addressing these issues can enhance the feasibility, sensitivity, and accessibility of screening programs, resulting in favorable impacts on the early diagnosis and survival of breast cancer on a global scale.

## Introduction and background

Breast cancer (BC) is a pervasive global health issue, affecting one in eight women and ranking as the second most common type of cancer worldwide [1]. According to World Health Organization (WHO) reports, the disease diagnosed 2.3 million new cases worldwide and claimed 670,000 lives annually in 2022. BC is a severe malignancy where abnormal cells within the breast multiply uncontrollably, forming fatal tumors. If left unchecked, these tumor cells can invade surrounding tissue and other organs like the lungs, bones, and brain, significantly impacting survival rates [2]. Treatments for BC are more effective and are better tolerated when detected early, improving patient outcomes [2,3]. Breast X-ray imaging programs, or mammograms, can identify suspicious lesions before signs and symptoms appear, also called screening (The Atlas of Breast Cancer Early Detection by the International Agency of Research on Cancer, 2023) [4]. Earlier detection is associated with better outcomes, lower morbidity, and reduced BC-related deaths [5]. Furthermore, populations with a reasonable uptake rate in screening programs can achieve a 90% five-year survival rate in patients who received an early diagnosis attributed to screening (WHO, 2021) [6]. Therefore, screening for BC is paramount, and routine mammography effectively reduces mortality from BC [5].

Breast imaging, or mammography, a specialized technique that uses low-dose radiation to detect changes in breast tissue, was first used in the early twentieth century after the discovery of X-rays. By 1985, it was the only method recommended for asymptomatic women with an average risk of BC. Since then, it has evolved significantly and remains the reference and adopted standard for detecting breast abnormalities, particularly those caused by early cancer [4].

Despite the recognition of BC as a global health concern, there are significant disparities in screening programs between developed and developing countries and even within a particular country. While developed countries allocate human and financial resources to increase BC awareness and develop national breast screening programs with mammography, developing countries often have limited public health resources. In addition, the presence of highly prevalent infectious diseases puts pressure on the allocation of resources in this direction [5,6]. Many developed or high-income countries have established nationwide and population-based breast screening programs with solid success rates, including Australia, the Netherlands, the United Kingdom, and the United States of America. For instance, in 2010-11, high-income nations like the United Kingdom's National Health Service Breast Screening Programme (NHSBSP), which screened 1.88 million women aged 50-70, reported a cancer detection rate of 7.8 per thousand women and 85% five-year survival for cancer patients. Australia, with the Breast Screen Programme, likewise saw a decrease in BC mortality rates since the program's inception, from 74 deaths per 100,000 women in 1991 to less than 50 deaths per 100,000 since 2010 [7].

Conversely, many low and middle-income countries (LMICs) face significant barriers that contribute to late-stage diagnoses and higher BC mortality. These include economic constraints, cultural stigma [7], and lack of high-quality health data sources, like population-based cancer registries [8], limited access to mammography facilities, lack of BC awareness and importance of screening, and inadequate distribution of treatment facilities [9]. Owing to this, international guidelines recommend self-breast examination (SBE) and clinical breast examination (CBE), low-cost screening methods, respectively, as an alternative in LMICs. However, it often leads to late-stage detection and poor treatment outcomes [10]. Many LMICs, such as India, Kazakhstan, Lebanon, South Africa, Uzbekistan, and Indonesia, are prioritizing the implementation of population-based mammography screening with the hope of reducing BC-related deaths [11]. While certain middle-income countries, like Brazil, have implemented national screening programs, access remains uneven, particularly in rural areas. The high costs associated with diagnosis and treatment further aggravate these disparities; for example, in Kenya, the average price of breast cancer diagnosis in the private sector is thrice the public health centers, making it unaffordable for many [7]. This trend highlights the exacerbating burden and the urgent need for targeted interventions to improve access and outcomes for BC globally.

On the other hand, the ability of screening mammography to detect BC effectively and early lies in guaranteeing its accessibility and performance at the highest level for all qualified patients. This systematic approach, known as quality assurance (QA), aims to achieve the maximum image quality with the least amount of radiation. Internationally, at least more than fourteen documents providing guidance have been developed specifically concerning mammographic QA [7,12]. Some countries, such as the United Kingdom (UK), have monitored quality through a national screening program since 1988 [13]. Many other nations have similar quality monitoring programs [12]. In Australia, the Breast Screen Australia National Accreditation Standards (BSANAS) have been in effect since 2015, and among a consortium of European countries, the European Guidelines for Quality Assurance in Breast Cancer Screening and Diagnosis (EGQABCSD), now in its fourth edition, have been pivotal in promoting adherence to standards ensuring the quality of screening and diagnosis services for breast malignancies [12,14]. However, in the United States (US), where thirty nine million mammograms are performed annually, there is no national screening program, but the federal government mandates minimum quality standards for the performance of mammography [12].

To sum up, many countries have developed BC screening programs and guidelines, particularly in developed nations, while others lack well-developed programs or have not yet implemented screening at all [15,16]. Even in nations with established initiatives, the examination coverage remains somewhat limited. This disparity emphasizes the urgent need for targeted interventions to enhance mammography quality assurance and establish uniform, comprehensive guidelines, ensuring equitable access to early detection and treatment of BC for all women, regardless of their socioeconomic status or geographic location [16].

Given these challenges, the purpose of this review is to examine the current practices of mammography screening worldwide, identify key disparities affecting the uniformity and potential research directions toward resolving these gaps, and act as robust evidence towards proposing efficient strategies for continually improving access and quality of BC screening on a global scale.

## Review

Epidemiological data

Precise epidemiological profiles are crucial for planning, executing, and assessing screening programs. These profiles provide essential baseline information, like disease incidence, population demographic data, healthcare system abilities, and screening programs' overall efficacy. Nevertheless, substantial obstacles exist in gathering and examining such data due to obvious resource and funding constraints in most developing countries [17,18]. Numerous countries confront difficulties in timely reporting and systematic registration of interval or hidden cancers (cancer cases detected outside the time frame between scheduled surveillance screenings), primarily due to fragmented healthcare systems or the lack of centralized cancer registries, skewing the perceived effectiveness of screening programs [19,20].

Moreover, present epidemiological assessments often lack the detail required to endorse personalized screening programs based on individual patient risk variables, such as family history, genetic predisposition, etc., emphasizing the need for more advanced risk-stratified data documentation [21,22]. These gaps have led to the development of more sophisticated models and strategies, such as the Global Cancer Observatory (GLOBOCAN), an online cancer forum whereby epidemiological data are collected and made available for comparison across countries to allow for decision-making [23]. However, their implementation is still limited, requiring standardized methods for data collection, personalized risk assessment, extended monitoring for interval cancers in suspected cases, and implementing uniform guidelines to enhance the effectiveness of screening programs [20,22]. International collaboration and information sharing may be critical in developing these strategies, especially in resource-constrained environments [24].

Radiographer qualification and training

According to the European guidelines for quality assurance in BC screening and diagnosis, a breast unit must have a core team composed of skilled healthcare professionals of various disciplines who have undergone specialist training in breast cancer beyond their general theoretical education [25]. Studies, however, report that most radiographers often experience a lack of confidence and adequate hands-on training, leading to truncated skills and challenges in providing accurate mammography screening [26]. Utilizing experienced and qualified radiographers in this field and training them to interpret mammograms can improve the quality of screening programs [27].

Qualifications and the training requirements for performing mammography screening for radiographers vary across the continents. In the United States, radiographers need to hold a state license or general certification from the American Registry of Radiologic Technologists (ARRT) together with mammographic certification. This basic framework guarantees a general level of preparedness [28]. On the other hand, certain European nations like Estonia, Finland, Norway, Portugal, and Switzerland provide training and continued professional development (CPD) programs, but recent studies demonstrate issues screening and radiographers face in terms of resources, time, and variability across the country to country within the continent. Researchers have shown that while these programs exist, there seems to be a need for other extensive mammography knowledge such as positioning of the breast and addressing the patient [26,29].

On the other hand, low- and middle-income countries (LMICs) such as India and Mexico provide a relatively different picture. Radiographers practicing in India must have a minimum diploma or degree in radiography and should have training in mammography. However, it can also be seen that there is an importance placed on practical experience, receiving degrees, and engaging in further education [30,31]. Mexico, for instance, provides short training with few components related to mammography, and most of the training is obtained through work experience [31]. This goes to show why training in these regions should comprise a general and standardized program.

Thus, to narrow these gaps and improve mammographic quality all over the world, coordinated actions with ongoing quality control, patient tracking, effective communication for patient follow-up, and provider feedback, all associated with significant and ongoing operating costs, are needed [26,32]. Promoting structured training with long-term assessment systems, as shown in some research findings, is essential to avoid the problems highlighted above [33]. Using tools such as the Perfect, Good, Moderate, Inadequate (PGMI) scale can improve image quality and satisfaction among radiographers [34]. Furthermore, expanding the role of radiographers in screening programs, such as interpreting the images, an approach implemented in the United Kingdom, can significantly increase cancer detection rates and improve service provision [35]. Across nations, comparative investigations are also critical to pinpointing the structure of the practices and developing a constitution of evidence-based policies [26].

Image quality parameters protocol

A standardized protocol accounting for various image quality characteristics remains imperative for mammography screening due to its reliance on technical conditions. While several European and American nations have made significant progress in standardizing image quality parameters by adopting globally approved protocols, many are yet to do so [25]. The absence of protocol uniformity lies in many countries, contributing to variations in image quality and cancer detection rates [26]. Metsala et al. revealed that European centers simultaneously used various protocols, ranging from locally, nationally, and internationally developed standards, challenging the clinical performance and cancer detection rate of screening mammography. They also emphasize that it leads to diverse practices in crucial factors such as positioning, image contrast, breast compression, interprofessional working, positioning sheet usage, and quality control programs, causing suboptimal clinical performance comparisons and screening practices [26]. Outdated international standards, particularly regarding positioning for fundamental views, further contribute to these issues, as highlighted by Sweeney et al. [36].

Therefore, establishing a comprehensive, internationally recognized image quality protocol is essential to address these technical intricacies of mammography screening. Such a protocol must include defined minimum technical requirements for mammography equipment with specified calibration requirements and frequency; guidelines for interprofessional collaboration [25]; healthcare provider expertise criteria including qualification and competencies [26]; specific image quality parameters like breast compression, image contrast, and noise with quantitative measures; comprehensive quality-control tests and audits methods [33], and standardized positioning techniques based on updated guidelines [36]. Implementation could involve forming an international task force of experts or collaborating with international organizations, conducting multicenter validation studies [26], and developing standardized training modules for radiographers [26,37]. The protocol should also incorporate distinct roles and responsibilities for the advanced practice of radiographers and a system of regular updates to keep pace with recent additions and advancements [36,37]. This will address the diverse practices currently observed worldwide, leading to improved cancer detection rates and patient outcomes.

Automatic exposure control (AEC) optimization

Automatic exposure control (AEC) is a faster and more convenient approach to adapting exposure to a patient in screening mammography. It can optimize two critical aspects: exposure parameter adjustment for a scan as per the breast thickness and density, and minimal dose exposure to the breast during screening. Manufacturers provide several AEC modes, allowing specific adjustments and customization on the image's contrast to noise ratio (CNR) and the minimal dose depending on the screening patient's characteristics [38]. However, despite these advancements, several barriers and obstacles hinder the consistent and optimal use of AEC globally. A recent European pilot study with five countries also demonstrated the heterogeneous AEC usage patterns among radiographers and found that half of the radiographers utilized manufacturer-specific dose-saving modes, while the other half considered multiple AEC modes [39]. The primary reasons highlighted were in line with other studies, that is, variable options for AEC were available, but lack of guidelines on the most optimal parameters and modes of AEC according to the requirement and setting, lack of comprehensive training on its usage leading to inconsistent application, different manufacturers providing numerous AEC modes with their own preferences, complicating standardization efforts, and creating obstacles for radiographers when switching between systems [39,40].

Research is ongoing to compare the effectiveness of AEC and manual optimization by radiographers. Some studies have found that manual optimization allows operators to account for a broader range of factors compared to AEC, such as breast size, composition, etc., and achieve similar image quality with lower radiation doses, especially for smaller breasts [41]. These findings suggest that while AEC provides a standardized approach, skilled radiographers' manual adjustments of exposure to a patient can sometimes surpass automated settings.

Thus, integrating AEC technology with radiographer expertise in fine-tuning and adapting these settings cannot be understated. To overcome these challenges, efforts should focus on standardizing AEC protocols across manufacturers and centers and improving radiographer education on AEC modes and their effects on dose and image quality [42]. Additionally, enhancing collaboration between radiographers, medical physicists, and radiologists to optimize technical parameters and conducting comprehensive studies to find the right balance between automation modes and technologist expertise are needed to maximize the benefits of AEC and achieve the best possible image quality with minimal radiation exposure during screening [39,42,43].

Target-filter combination

The target and filter combination choice significantly affects mammography's image quality and radiation dose [42,44]. Most commonly, tungsten or molybdenum targets with rhodium, silver, tin, and molybdenum filters are available, which, on an optimal selection, can reduce glandular dose to the breast during screening. Selecting the optimal target-filter combination for each patient can be complex, as it depends on various factors such as breast thickness, density, and composition. There's a lack of consensus on which combinations are best suited for different breast types, highlighting the need for more comprehensive research in this area [45,46].

Several studies have investigated the performance of different target-filter combinations. Nunes et al. reported that tungsten-rhodium (W-Rh) combinations produce better image contrast and visibility across breast thicknesses and densities at lower radiation doses. They also noted that this combination maintains mean glandular dose (MGD) by 30% compared to the traditional combinations, thereby rendering it the optimal choice for breast imaging, especially for thinner breasts [47]. Similarly, Ghomalkar et al. observed that the W-Rh reduced MGD by 60% compared to the tungsten-silver (W-Ag) combination at low exposure settings while also offering superior signal-to-noise ratio (SNR) and contrast-to-noise ratio (CNR) [48]. However, these studies had a small sample size or specific imaging conditions like exposure settings and equipment used, thus affecting their generalizability.

The performance of molybdenum-based combinations has reported contrasting results, adding further complexity with mixed results. Varjonen et al. and Izhidar et al. found that molybdenum-molybdenum (Mo-Mo) and molybdenum-rhodium (Mo-Rh) combinations provide reasonable image quality but do not achieve the same level of dose reduction as W-Rh. Their findings support the conclusion that W-Rh is superior in reducing MGD while maintaining high image quality [44,49]. Others, like Aminah et al., demonstrated that Mo-Rh may be suitable for specific breast thicknesses, but the W-Rh combination outperforms it across a broader range of breast compositions [43]. Additionally, Biegala et al. reported a significant 23.6% reduction in MGD with W-Rh compared to Mo-Mo and Mo-Rh combinations, emphasizing its practical benefits in clinical settings [50]. These studies focused on comparing only a few named combinations and different evaluation methodologies, leaving gaps in our understanding of how these combinations perform in various conditions.

Although W-Rh demonstrates promising results, the current evidence base is insufficient, with limited comparative evaluations of target filter combinations to make universal recommendations. More large-scale multivariate studies comparing all available combinations, evidence-based guidelines for selecting target-filter combinations considering patient-specific factors, and specialized training for radiographers and medical physicists are consequently warranted [45,51].

Repeat and reject film analysis

Repeat and reject analysis is a vital quality control measure in mammography screening, evaluating images that are discarded or repeated due to various technical or quality issues. Despite the transition to digital systems for screening, image rejection and repetition rates still exceed established standards, leading to increased radiation exposure, resource utilization, cost, and patient discomfort [52,53]. A study by C. Prieto et al. at a university hospital evaluating the retake rates in digital mammography found that central issues for image rejection were incorrect positioning, closely larger breasts requiring multiple exposures, use of small flat panel detectors, and other technical reasons [54]. This problem has previously been demonstrated in studies with screen film, computed radiography, and, more recently, digital breast tomosynthesis [52,54,55]. Conversely, studies have also shown that radiographers without specific mammography qualifications rejected more images than those with specialization [52].

Healthcare facilities in many countries have enforced quarterly analysis, log maintenance, planning training sessions, and the correct use of technique charts, improving optimal repeat and recall rates and elevating screening outcomes. Challenges persist regardless, due to practice variability and the absence of universal guidelines [53,56]. To address this challenge, widespread implementation of schemes such as quarterly analysis, standardized reporting systems, training sessions, and ensuring appropriate repeat and rejection criteria selection can encourage effectiveness and research on screening programs [53,54].

Breast compression methods

Proper breast compression methods during patient positioning are widely incorporated and indispensable for enhancing detection sensitivity in mammography screening. It helps reduce breast tissue overlap, reducing radiation dose, and improving image quality [57]. Unfortunately, present methods lack standardization and reference standards to evaluate compression force uniformity, contributing to a patchwork approach among practitioners and healthcare institutions. Moreover, radiographers often rely on their own experience and subjective interpretations of the established recommendations when employing breast compression, limiting mammographic examinations' reproducibility [58,59].

Recent research establishes that compression pressure, measured in kilopascals, can be a more reproducibly reliable, physiologically relevant, and patient-specific parameter than force for a uniform application of breast compression. It can account for the force applied, the contact area of the breast, breast size, and stiffness differences while reducing patient discomfort and radiation exposure compared to compression force, increasing the willingness of women to participate in screening programs [59,60]. This pressure-based standardization protocol has also demonstrated potential efficacy in improving image quality and diagnostic yield. Nonetheless, there is an urge to conduct evidence-based comparative surveys on compression force and pressure-guided approaches, seeking patient feedback and establishing relevant future breast compression strategies [60,61].

Glandular dose estimation methods

Mean glandular dose (MGD) or average glandular dose (AGD) is an essential parameter that governs the risk versus benefit of screening mammography and refers to the glandular dose absorbed in the breast tissue [24]. Nevertheless, inconsistencies arise in the evaluation of MGD due to the difference in the breast characteristics, density, and composition, varied measurement techniques used by different mammography system vendors such as Philips, Fujifilm, Hologic, and General Electric (GE), and lack of comparative evaluation of these techniques [62]. This subjective approach leads to global disparity among radiographers, underlining the requirement for higher quality assurance for dose estimation methods [63].

Several investigations are exploring new methods and metrics that can be feasibly standardized and provide accurate dose estimation, including modifications to the traditional MGD estimation and breast-specific dose models that can be conditioned with country-specific variability of breast tissue density and their sensitivity to radiation [64,65]. Furthermore, a recent metric, average absorbed breast dose (2ABD), has been proposed as a potential replacement for typical MGD assessment, with a 2017 study depicting it as more precise and effective in reporting doses [66]. However, extensive research is needed to substantiate a uniform dose estimation method by comparing estimated doses to actual measurements, evaluating the new metrics, developing breast characteristics-specific dose models, conducting regular dose estimation audits, and collaborating to standardize processes across different vendors [65,67].

Screening frequency

Robust quality control recommendations for screening frequency have been shown to significantly reduce mortality rates by up to 30% and increase the efficiency of mammography [68]. However, its guidelines are sometimes diverse and conflicting, with most guidelines having a consensus that mammography is the gold standard screening modality for average-risk women. For this risk group, most of the guidelines suggest annual or biennial mammographic screening at 40-74 years, while screening should particularly focus on 50-69 years [68,69]. For instance, the American Cancer Society (ACR) recommends annual screening starting at age forty-five. In contrast, the National Comprehensive Cancer Network (NCCN) and American College of Obstetricians and Gynecologists (ACOG) recommend yearly screening starting at age forty, and the Society of Breast Imaging (SBI) states that women should begin annual screening at forty and continue until their life expectancy reaches five to seven years [70,71]. This lack of uniformity in recommendations, especially for women in their forties, creates confusion and potentially impacts screening adherence [69].

To address these challenges, several potential solutions are being explored, such as standardizing screening recommendations based on the latest evidence and ongoing research to better understand the balance of benefits and harms of mammography screening, particularly in younger women [68]. Efforts are also being made to improve adherence to screening recommendations through patient education, provider training, and addressing barriers to the feasibility. Nevertheless, more studies assessing the impact of screening intervals on mortality and false-positive rates, developing risk-group-based protocols, improving patient education, and international conferences can help harmonize screening recommendations [71,72].

Resource constraints

LMIC breast cancer incidence is lower than in high-income countries, despite higher mortality rates due to apparent resource constraints than in high-resource nations [73-75]. Olivera Ciraj-Bjelac et al. and Erkin Aribal et al. identified some of these resource challenges, which include a lack of trained personnel, poor quality control facilities, and compromised access to essential supplies for screening. These limitations compromise the feasibility of implementing large-scale screening programs, exacerbating the existing dearth of awareness about breast cancer among the general population [76,77].

Some LMICs have adopted alternative screening strategies that may be more realistic and cost-effective, such as clinical breast examination with targeted ultrasonography, reducing the uptake of mammography screening, but this cannot be stated as the definitive solution [78,79]. Hence, to prioritize breast cancer screening in low- and middle-income countries, it is prudent to concentrate on more dependable interventions such as executing breast health awareness campaigns, establishing collaborations with affluent nations, and using telemedicine and mobile screening units in underserved regions [75,80].

Diagnostic reference levels

Diagnostic reference levels (DRLs) are a highly effective quality assurance tool for optimizing doses in radiological examinations across different countries and institutions. They provide a benchmark for comparing facilities, adjusting their dose levels, and identifying trade-offs [81-83]. Currently, DRLs are determined using the third quartile values of mean doses observed on a sample of standard-sized patients in each radiography room, which allows for the identification of abnormally high doses and the establishment of national DRL values; however, this method is only applicable to systems from specific manufacturers, leading to inconsistencies that make comparisons between countries and facilities difficult. Additionally, variations exist in data collection methods (patient data or phantoms), and calculating the doses and infrequent updates of DRLs further complicate reliable DRL establishment and its optimized practices [82,84].

In certain nations, DRLs are being regularly implemented and updated, with studies stating that with their use, radiation doses could be significantly reduced [85]. Despite these efforts to establish international consensus on DRL protocols and methodologies for mammography, an in-depth inquiry into patient-specific DRLs, standardizing protocols, data collection procedures, and instituting a universal dose calculation method are warranted [86]. Finally, promoting a systematic approach for regular DRL review updates and facility participation in national and international dose surveys can improve radiation dose optimization during screening [38,86].

## Conclusions

This review highlights several critical areas that need improvement in mammography screening programs globally. Some major obstacles involve diverse approaches for data collection requirements in epidemiology, mainly in developing nations; training and qualifications of radiographers; setting standardized protocols for image quality and breast compression; optimizing exposure control and target-filter combinations; analysis of repeated and rejected images; methods for estimating doses; recommendations for screening frequency; dealing with limited resources in low- and middle-income countries; and establishing uniform diagnostic levels.

Despite several advancements in screening programs, including updated frameworks, certified mammography radiographers, and new standards for dose measurement, there are still hurdles that the current methodology has not overcome. To maximize the efficiency and benefits to the patients, more studies are required to formulate guidelines for standardization and detailed protocols for initial data gathering, image quality parameters, and estimation of the dose. Additionally, further investigation is needed on identifying optimal screening frequencies for different population subgroups, defining guidelines for practice in low-resource environments, enhancing instruction of radiographers on proper routine protocols, and the integrated utilization of automated and manual control modes. More exploration is also required to establish the relevant target-filter combinations, breast compression strategies, and methods of dose assessment. Such endeavors, along with international cooperation to overcome the resource limitations in low and middle-income countries, will pave the way for devising better screening policies, enhancing image quality, reducing hazards, and leveraging breast screening and patient care globally.
